# Inhibition of Carbide Growth by Sr in High-Alloyed White Cast Iron

**DOI:** 10.3390/ma15041317

**Published:** 2022-02-10

**Authors:** Malwina Dojka, Rafał Dojka

**Affiliations:** 1 Department of Foundry Engineering, Silesian University of Technology, 7 Towarowa Street, 44-100 Gliwice, Poland; 2 Odlewnia RAFAMET Sp. z o. o., Staszica 1, 47-420 Kuźnia Raciborska, Poland; r.dojka@odlewnia-rafamet.pl

**Keywords:** white cast iron, crystallization, modification, wear resistance

## Abstract

Chromium cast irons have gained a well-settled position among wear-resistant materials where a low manufacturing cost is one of the key factors. The wear properties of these alloys are commonly improved by the addition of carbide-forming inoculating elements such as Ti, V, B, etc., allowing the formation of underlays for the precipitation of both M_7_C_3_ carbides and austenite. On the other hand, Sr may work as a surface-active element that adsorbs on the surface of the growing crystal, inhibiting its growth. This mechanism may support the M_7_C_3_ nucleation process. The experiment was conducted on near-eutectic chromium cast irons with 20% of Cr and 2.5–3% of C. Different amounts of strontium were used as the microstructure modifier. The improvement of carbides’ stereological parameters and collocation resulted in the improvement in functional properties—wear resistance and impact strength without a significant increase in hardness as well as a decrease in carbide phase. Two types of wear studies with a modified pin-on-disc method and tests in reciprocating motion of samples in the metal-mineral system were performed. The results showed that addition modification with Sr can increase the impact strength of the alloy. EDS analysis of the samples provided results similar to hypoeutectic Al-Si alloys modified with strontium.

## 1. Introduction

**Figure 1:**
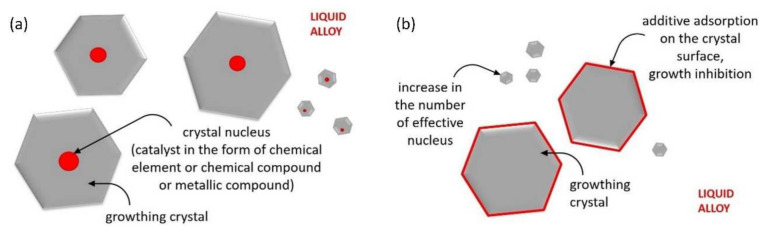
Schematic diagram of the effect of (**a**) nucleogenic elements on the growth of crystals [1]; (**b**) surface-active elements on nucleation and the growth of crystals.

There is little information available in the literature that refers to the effect of surface-active elements on the structure and the properties of chromium cast iron. Most of the literature sources describe the effect of nucleogenic elements, especially carbide forming elements such as Ti, Nb, V, and W. Typically, they are added to the melt in the form of ferroalloys. They create hard-to-melt compounds (TiC, NbC, VC, etc.) that serve crystallization underlays for primary austenite and chromium carbides. The influence of this type of addition was also discussed in previous works, which suggested that the inoculation of those types of alloys may result in the formation of bifilms [1] as well as the segregation of crystallization underlays [2,3]. Figure 1a presents the diagram of nucleogenic modifier operation [1]. This mechanism allows microstructure refinement, increasing the number of eutectic colonies (γ + M_7_C_3_) and the obtainment of improved carbide morphology. Inoculated chromium cast iron usually achieves better mechanical properties and superior abrasive wear resistance. However, hard carbides formed in the alloy microstructure may induce an increased tendency to crack [4,5]. The operation diagram of surface-active modifiers is shown in Figure 1b.

By comparing Figure 1a to Figure 1b, we can notice a difference between the action of nucleogenic and surface-active elements. The surface-active elements stimulate the growth of crystals from the outside as opposed to the nucleogenic elements whose presence can be noticed inside the growing phases. The theories concerning the surface-active modifiers or simple modifiers dissolved in alloys have been found to be true mainly for non-ferrous metals. The impact of elements including strontium, sodium, or antimony has been described in detail for silumins or other alloys, e.g., magnesium alloys. Thus, the modifying effect of the above-mentioned elements manifested by grain fragmentation and enhanced mechanical properties has been confirmed. Researchers described [6,7,8,9] that a small amount of strontium or sodium in aluminum-silicon hypoeutectic alloys affects eutectic Si phase transformation from a plate-like (flake) structure to a fine fibrous (coral-like) structure. Timpel et al. [10] established that the trace Sr addition in Al-Si alloy may significantly change the morphology of the eutectic Si phase. The Sr-Al-Si phases segregate and restrict the growth of Si crystals. Studies of Al-Si alloys properties [11,12,13] reveal that strontium additives may increase the ductility and strength of modified material. Research by Chen et al. [14] showed that the simultaneous addition of Sr and Y helps to obtain fine grain microstructure in AZ31 magnesium alloy, while at the same time, the additions improve corrosion resistance of examined material. Han et al. [15] proved that Mg-6Al magnesium alloy with Sr exhibits superior mechanical properties in comparison to the alloy without strontium as a result of microstructure fragmentation. A different situation is observed for grey cast iron. According to the research of Stefanescu and Riposan et al. [16,17,18,19,20], Sr, as with many other elements (Al, Ca, Zr, Ba), forms complex oxides and sulfides that act as a crystallization underlay for fine flake and nodular graphite. Therefore, in this case, we are dealing with an inoculation process. Strontium in grey cast irons supports the nucleation of graphite.

Nevertheless, no reference to high-alloy cast iron is available, which is worth analyzing. This is especially important in order to avoid brittle cracks in chromium cast iron that are sometimes related to the presence of additional hard carbides created by frequently used nucleogenic additives. For example, the negative impact of Ti addition on selected mechanical properties and casting defects was presented in the author’s previous work [1].

**Figure 2:**
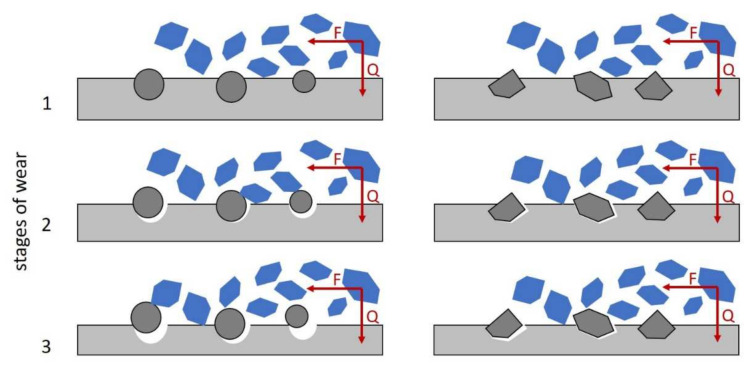
Three consecutive stages of abrasive wear for material with fine spherical carbides and material with fine irregularly shaped carbides.

The great importance during cast iron manufacturing is attached to creating M_7_C_3_-type carbides in the structure that feature high hardness and abrasive wear resistance. The objective of some experimental works is to obtain the most rounded shape of carbide precipitations [21,22,23,24,25]. The spheroidal carbides have a minimum possible expanded surface area. This could reduce the internal notch and related strains, and can also translate into higher cast iron strength. However, the metallic matrix plays a certain role in shaping the properties of chromium cast iron. Each alloy, depending on its application and intended use, should show a specific carbide inclusion morphology to extend the life of casting under conditions assumed. Moreover, spherical hard-phase precipitations chip from the matrix more easily (see Figure 2).

**Figure 3:**
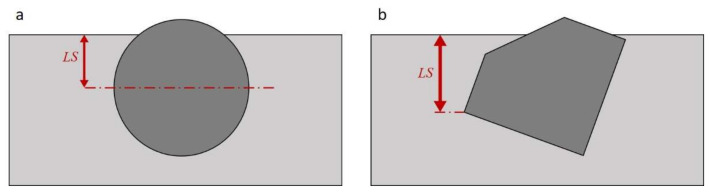
Matrix wear limit size *LS* allowing for maintaining the particle: (**a**) spherical one; (**b**) example irregular one.

Blue abrasive erodes the grey matrix of the alloy. For hard spherical inclusions, the limit wear size for metallic matrix *LS* (see Figure 3a) that allows retaining the particle will adopt the maximum values approximately equal to the radius of a specific spherical inclusion. For irregularly shaped carbides, the limit dimension *LS* can be higher (see Figure 3b).

The great importance of the presented work was learning how to enrich high-chromium cast iron by adding surface-active modifiers and assessing their effectiveness. The conducted investigations were supposed to confirm the opportunity to use selected surface-active modifiers to improve the morphology of the carbide phase and the tribological properties of high-chromium cast iron. Therefore, it was assumed that the modification of high-chromium cast iron with strontium can change the mode of alloy crystallization by absorbing strontium on crystal boundaries and by inhibiting the growth of chromium crystals. This results in creating uniformly fragmented carbide eutectics, improving the impact strength and abrasive wear resistance of the alloy without introducing additional carbide phase as in the case of commonly used inoculation, which negatively affects the impact strength.

## 2. Materials and Methods

**Figure 4:**
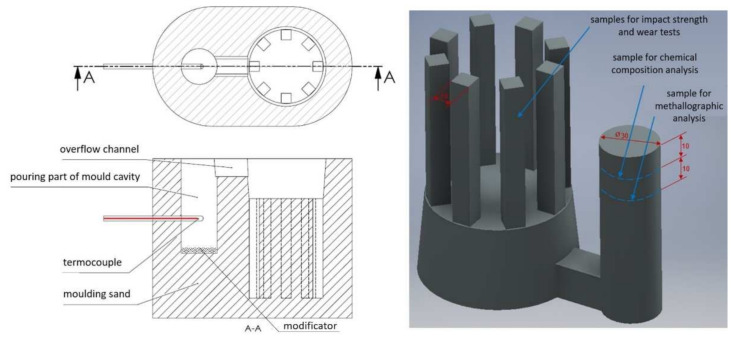
Scheme of mold cross-section (ATD-P tester); casting 3D model [1,4].

The examined material was 20% chromium cast iron with a variable amount of strontium additions. To verify the assumption, the experimental melts of chromium cast iron were made. All the melts were performed in the laboratory of the Department of Foundry Engineering, Silesian University of Technology in a medium-frequency induction furnace (PI25, ELKON Sp. z o.o., Rybnik, Poland) with a capacity of 25 kg. The presented work is part of a project implemented in the department [1,2,3,26]. The experimental castings were obtained by filling the ATD-P tester mold described in other authors’ work [1,26]. The scheme of the mold and model of the casting with marked sampling places for individual tests are presented in Figure 4. Charge materials were chromium cast iron ingots; during the melting process, a carburizer was added to obtain a near-eutectic chromium cast iron composition. Metal was poured into the ladle when the furnace temperature reached 1550 °C. Every melt was deoxidized with Al (0.1% by weight of charge) placed on the bottom of the preheated ladle. The metallic strontium was added to the mold by placing it on the cavity bottom, as shown in Figure 4. Table 1 presents the chemical compositions of experimental samples obtained from the spectrometric analysis by using the LECO GDS500A spectrometer (Model No607-500, LecoCorporation, 3000 LakeviewAve, St. Joseph, MI, USA). The Sr addition was calculated for the weight of casting as: 0.05%, 0.1%, 0.15%. After the casting process, the samples were prepared for tests.

Metallographic studies were conducted by using light microscopy and scanning electron microscopy (SEM). The microstructure of experimental samples was analyzed with a Nikon light microscope (Eclipse LV150N, Nikon Metrology Europe NV, Geldenaaksebaan 329, Leuven, Belgium). The samples were prepared by wet-polishing using sandpapers, buffing, and etching in ferric chloride solution for about 15 s. The microstructure analysis also included the examination of the chromium carbide stereological properties using NIS Elements software (NIS-Elements Advanced Research, Nikon Instruments Inc., 1300 Walt Whitman Road, Melville, NY, USA). SEM analysis with the Phenom ProX (Phenome-World Eindhoven, North-Brabant, the Netherlands) was carried out on unetched metallographic samples.

Rockwell hardness and impact tests were performed on samples with a square cross-section (see Figure 4). The Charpy impact test was executed by using a SUNPOC Impact Tester (JB-300B Pendulum Impact Testing Machine, GUIZHOU SUNPOC TECH INDUSTRY CO., LTD., Guiyang, Guizhou, China). At least three measurements of hardness and impact strength per sample were conducted. The fracture surfaces of the impact tests samples were analyzed.

The abrasive wear-resistance tests in reciprocating motion and by using the pin-on-disc method were carried out on prototype devices designed in the Department of Foundry Engineering in Gliwice [27,28]. Wear analysis was executed on square cross-section samples (see Figure 4); the surface area of the sample facing the abrasive was 100 mm^2^. The abrasion processes were executed under the same conditions as they took place in previous studies [3]. For both methods, the dry abrasion process was followed. In the pin-on-disc test, the abrasive disk was sandpaper P80 with aluminium oxide. The rotational speed of the abrasive disk was 155 RPM; the rotational speed of the sample holder was 400 RPM. The loading per sample was 220 g. Before the test, each sample was pre-abraded to provide a full-face contact area with the abrasive material. Each sample was then weighed, and the initial weight was determined. To start the test, the abrasive disk was replaced with a new one before starting a new test series. The sample was placed in the holder on a rotational head. The test consisted of performing six 10 min test cycles. After each test cycle, the sample was cleaned, and then it was weighed to check for any loss in weight during abrasion. For the test in reciprocation motion, the sample was placed in a holder on the arm driven by a 3-phase electric motor. Below the tested material, a SiC abrasive sandpaper P50 was attached as the counter sample material. The sample face was loaded with a preset load of 10 N. The samples during the motion were subject to abrasive wear as a result of friction. Samples were pre-abraded at first. The abrasive wear test started by weighing all samples on a laboratory scale. Then, the weight was attached (a 1 kg disk) and the machine was set on 1000 cycles of motion. The whole measurement included 5000 motion cycles, which provided the total sample travel of 1000 m in a reciprocating motion. After every 1000 cycles, the abrasive paper was replaced. At the end of the measurement, each sample was weighed and the loss in weight was calculated. The abrasive wear test was repeated at least three times for each sample.

## 3. Results

### 3.1. Microstructure Analysis

**Figure 5:**
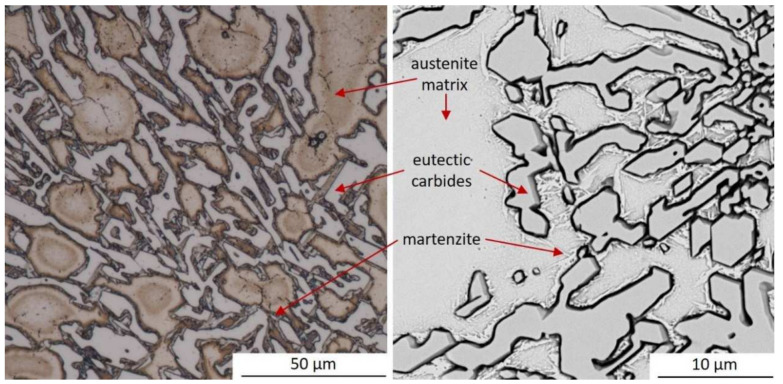
Micrographs from optical microscopy and SEM for as-cast experimental chromium white cast iron.

Figure 5 presents optical microscopy and SEM micrographs of the examined as-cast chromium cast iron with phases marked. The microstructure of the samples consists of eutectic M_7_C_3_ carbides in an austenitic matrix. At the carbide–matrix boundaries, the martensite formed, which, according to other researchers [29,30], may be a result of C and Cr depletion in this area.

**Figure 6:**
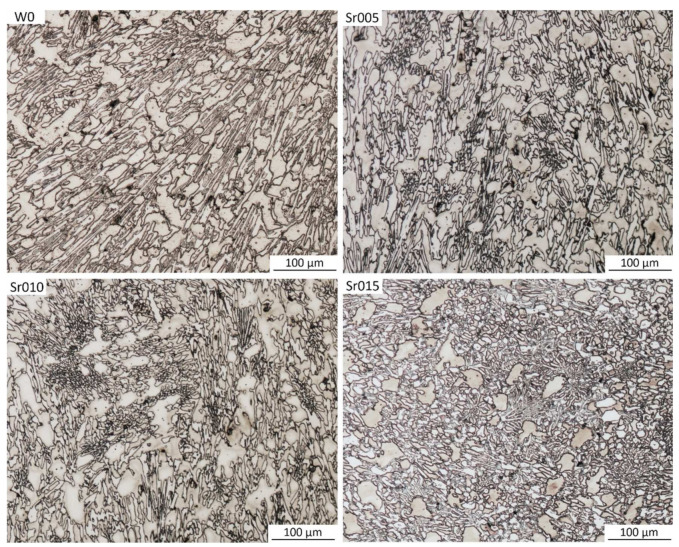
Micrographs from optical microscopy for samples: without strontium addition (W0); with 0.05% of Sr addition (Sr005); with 0.1% of Sr addition (Sr010); with 0.15% of Sr addition (Sr015).

Figure 6 shows the comparison of four examined samples: without Sr and with 0.05, 0.10 and 0.15% of Sr.

It can be noticed that the W0 sample presents Cr carbides with a needle-like shape. Modification with Sr allows to refine the microstructure and increase the number of eutectic colonies. Additionally, the microstructure of the W0 sample is highly directional. It can be seen that the higher the amount of added Sr, the less directional the microstructure becomes.

**Figure 7:**
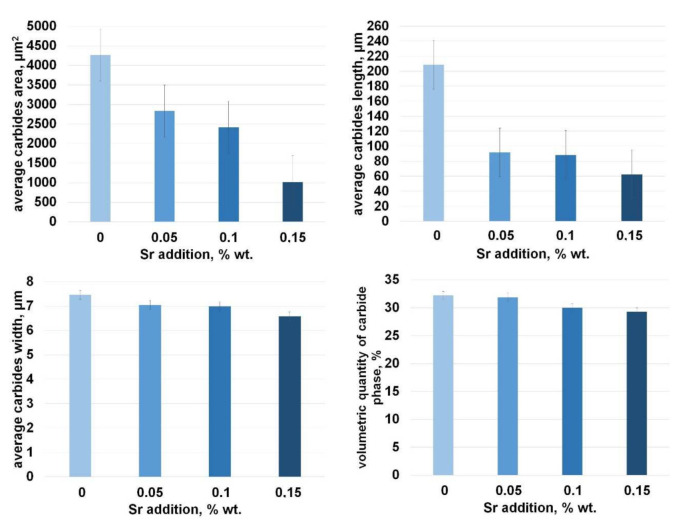
Selected stereological parameters of chromium carbide analysis in a function of added Sr.

For all samples with strontium, an improvement in microstructure can be seen—the majority of longitudinal, sharp-ended carbide inclusions observed for the sample without additives was replaced by γ + M_7_C_3_ eutectics rich in finer chromium carbides. In the samples with strontium added, we cannot notice any considerable growth in primary austenite dendrite arms and any reduction in the carbide phase share. In Figure 7, selected stereological parameters of chromium carbide analysis results are presented.

By analyzing the influence of strontium on the value of length and carbides area, it can be clearly noticed that the parameters related to the size of carbides such as their area, length, and width decrease as the amount of strontium increases. The addition of 0.05% of Sr decreased the average area of carbides by more than 30%, the addition of 0.1% Sr brought a further decrease of more than 40%, and 0.15% of added Sr resulted in a more than 75% decrease. For 0.05 and 0.1% of Sr, the average length of carbides decreased by 55%, whereas for the sample with 0.15 of Sr added, a huge, over 70%, decrease in length was observed. What is quite interesting and valuable is that if we look at the volumetric quantity of carbide phase, it can be seen that it does not decrease much with an increase in Sr addition.

**Figure 8:**
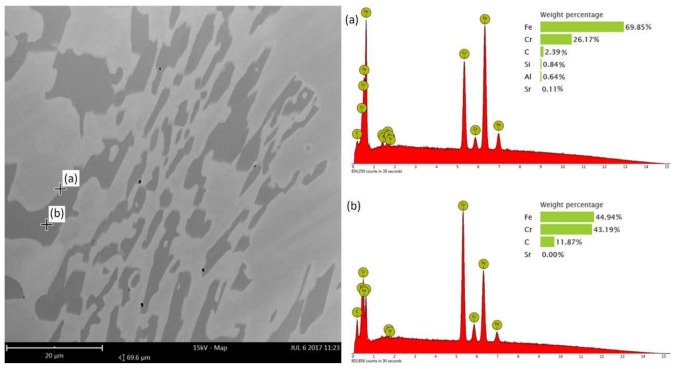
EDS point analysis on the border austenite–chromium carbide (**a**) and inside carbide (**b**) in Sr005 sample, SEM.

An energy-dispersive spectroscopy (EDS) analysis of chemical composition was performed; the point analysis (Figure 8) of the microstructure of the alloy modified by adding 0.05% of Sr shows that the concentration of strontium increases at the boundary of M_7_C_3_ carbide-austenite, while strontium does not occur in the very carbide.

### 3.2. Impact Strength and Hardness

**Figure 9:**
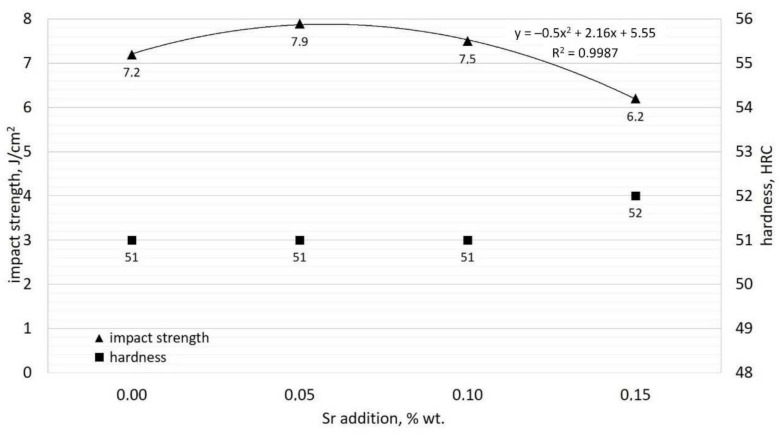
Influence of Sr addition on impact strength and hardness of examined alloys.

For the examined group of chromium cast iron samples, it is possible to determine a binomial dependence for the impact strength change in the function of modifying the additive quantity. Figure 9 presents this dependence for the alloy in the function of strontium addition quantity. In Figure 9, there are also marked values of Rockwell hardness for the examined alloys.

The highest impact strength, by almost 10% higher than the initial alloy, was shown by chromium cast iron with 0.05% of Sr added. When using 0.1% of strontium, the impact strength decreases; however, it is still higher than for the alloy without Sr added. The use of 0.15% of modifier in the form of strontium resulted a reduction in the impact strength as compared to the initial cast iron. The value of the R^2^ determination coefficient indicates a connection between the impact strength value and the quantity of modifying elements added. In terms of improving the impact strength, the most effective seems to be the modification with strontium at a quantity ranging from 0.05 to 0.1%. Samples with 0.0, 0.05, and 0.10% Sr present a hardness of 51 HRC; the sample with 0.015% Sr added obtained a slightly increased hardness of 52 HRC. This indicates that added levels of Sr have minor or no impact on the hardness of the alloy.

### 3.3. Wear Resistance

**Figure 10:**
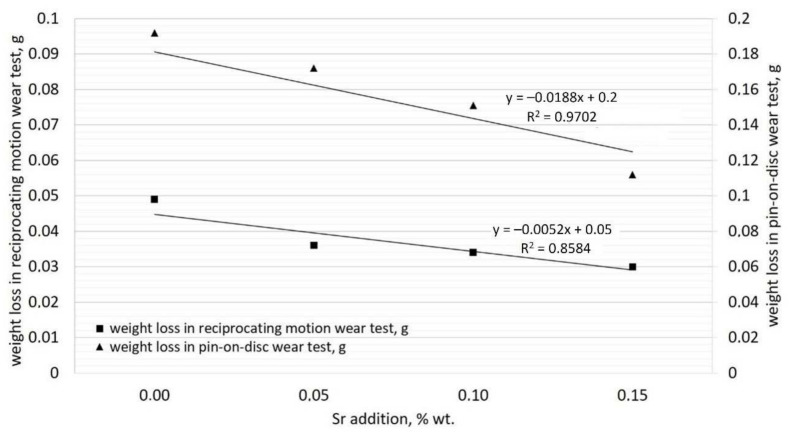
Influence of Sr addition on wear resistance of examined alloys.

As shown in Figure 10, Sr addition positively influences the wear resistance of the alloy. This could be the effect of the refined carbides in samples with added Sr, which could be less prone to being torn out from the matrix in reference to needle-like carbides in the unmodified alloy.

We can observe a dependence between the loss of weight and the quantity of Sr modifying addition. With an increase in the Sr level, the loss of sample weight during abrasion reduces as compared to the sample containing the initial cast iron (W0).

## 4. Discussion

**Figure 11:**
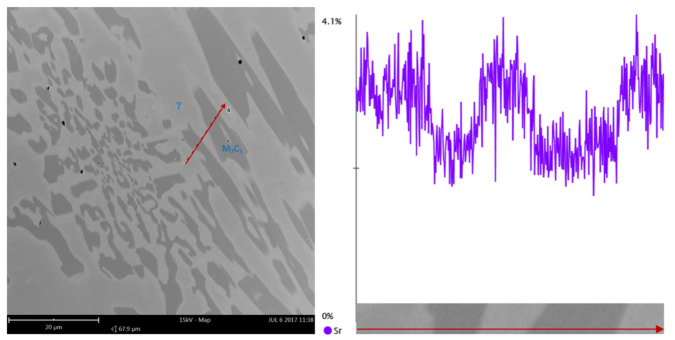
EDS linear analysis of Sr level in the eutectics of chromium cast iron sample with 0.005% Sr addition, SEM [2].

The presented analysis may lead to the conclusion that strontium has a different impact on the crystallization of chromium cast iron than commonly used nucleogenic inoculants. The microscopic studies show that Sr can refine the microstructure of chromium cast iron, but there are some differences between its action and other elements that have already found application as inoculants for this material. The modification of high-chromium cast iron with strontium affects the way of casting crystallization, while the analysis of quantitative and qualitative microstructure tests show that strontium can be adsorbed at the boundary of chromium carbides, thus inhibiting their growth. There were no compounds created with Sr found in the microstructure during the metallographic studies. Previous works have shown that the addition of nucleogenic elements significantly decreases the volumetric quantity of carbide phase [1]. The analysis conducted for strontium seems to show different results. The average volumetric quantity is lower for samples with strontium, but it is not a markable decrease: for the highest amount of added Sr, the amount of carbides dropped from 32 to 29%. The EDS results presented in Figure 8 were also confirmed by the authors in their previous research, where identical results were obtained from the linear analysis by eutectic of chromium cast iron with Sr (Figure 11). It shows how the quantity of strontium increases in the eutectics when the eutectic carbide boundaries are exceeded, which is consistent with the mechanism of surface-active modification.

In the pin-on-disc test, the loss of weight for Sr015 sample was decreased by more than 40% in reference to the unmodified sample. Those results surpassed similar high-chromium cast iron inoculated with 2% Ti [31] as well as a composition of Ti and REE [3]. With the increased amount of added Sr, the weight loss of the sample lowers linearly; a straight-line regression with a high R^2^ coefficient was formed. This allows to state that considerable refinement of eutectic carbides by Sr is possible, which was observed in microstructure tests and the carbide phase stereology. For the reciprocating motion wear tests, a similar relationship was observed. An increasing amount of added Sr resulted in a linear decrease in sample weight loss: for the Sr015 sample, the difference was 40%. It seems that the results of two types of wear resistance tests are incoherent. What is unfortunate is that the Sr015 sample has the lowest impact strength. It may be connected with the high affinity of Sr to the air surrounding the melt during the mold filling. The formation of a large number of nonmetallic inclusions such as strontium oxides during the filling might have affected the properties of the alloy [32].

It would seem that while applying Sr for chromium white cast irons, the precise control of the pouring temperature is crucial. The boiling point for Sr is 1381 °C, which is why it is recommended to use Sr as an in-mold modifier. If used as an on-stream modifier, Sr should be applied during the mold filling instead of during tapping a furnace. For Sr to work as a surface-active element its dissolution in the melt is necessary; reaching the boiling temperature may result in an effect similar to Mg treatment during the cast-iron nodularization process.

Additionally, during in-mold modification, a Sr bearing modifier should be placed in an area with minimized turbulence. Increased turbulence results in the maximization of forming a large free surface of the flowing metal which is covered by oxides and silicates [33]. In order to minimize the turbulence during mold filling naturally, pressurized gating systems should be used [34]. The Gibbs free energy of Sr oxides and silicates is extremely low; the presence of pure Sr in the proximity of such compounds results in their reduction and precipitation of Sr compounds, which lose the ability to become crystal growth inhibitors. Such Sr action is proposed by Riposan [18,19] and Stefancescu for grey cast irons [16,17].

## 5. Conclusions

The presence of the martensite between the carbides and the matrix serves in a negative way as a buffer between hard carbide and plastic austenitic matrix phases. Under dynamic loads, for instance, during Charpy testing, the presence of martensite hinders the alloy from plastic deformation. Without the presence of martensite, during the load, the matrix could deform around the carbides; thus, the impact strength of the alloy would be higher. Future research should focus on the possibility of martensite elimination from the alloy.

With the addition of a certain amount of Sr, it is possible to increase the impact strength of the high chromium white cast iron by the refinement of chromium carbides as well as the improvement of their stereological parameters.

The addition of Sr allows increasing the wear resistance of the analyzed alloys. With 0.015% of added Sr, the wear resistance was decreased by around 40% in both pin-on-disc and reciprocating motion tests. In the pin-on-disc method, for 0.1% added Sr, the wear resistance decreased by 20%, and 10% for 0.05% of Sr. In the reciprocating motion test, for 0.1% added Sr, the wear resistance decreased by 30%, and 26% for 0.05% of Sr. This was reached without a significant increase in hardness.

Especially during market instabilities caused by the COVID-19 pandemic, the concept of critical raw materials (CRM) should be analyzed and kept in mind. The analyzed modification with Sr requires much less of the modifier in reference to traditional inoculation [3,31].

Castings that are manufactured from chromium white cast iron in most cases need to be replaced, not because they completely wear out but because of the cracking. That is why the application of inoculation modifiers such as Ti, Nb, V, or W can be problematic, as their use increases the hardness of the alloys. The surface-active modification allows to obtain grain refinement and an increase in the wear resistance without an increase in the hardness of the alloy. Such an approach, common in the case of Al alloys, is quite rare in the case of cast irons. The results of the conducted studies show that there is great potential for the application of this type of modification to improve the durability of wear-resistant castings. This is why in future research more surface-active modifiers will be tested to obtain the possibility of increasing the wear resistance of chromium white cast iron without increasing its hardness.

## Figures and Tables

**Table 1:** Chemical composition of experimental samples, % weight (spectrometric analysis), bal-balance.

	C	Cr	Ti	Mn	Si	Ni	Mo	Al	V	Zr	S	P	Nb	Sr cal.	Fe
W0	2.85	20.4	0.01	0.39	0.66	1.48	0.57	0.22	0.13	0.24	0.02	0.05	0.07	0	bal
Sr005	2.98	19.7	0.01	0.37	0.68	1.48	0.58	0.20	0.12	0.27	0.02	0.05	0.08	0.05	bal
Sr010	3.07	19.6	0.01	0.35	0.70	1.49	0.58	0.20	0.12	0.27	0.02	0.05	0.08	0.10	bal
Sr015	3.06	19.8	0.01	0.37	0.68	1.48	0.61	0.21	0.13	0.25	0.02	0.05	0.08	0.15	bal

## Data Availability

The datasets generated and analyzed during the current study are available from the corresponding author on reasonable request.
